# Heroic Treatment of Hysteria

**Published:** 1830-10-01

**Authors:** 


### HEROIC TREATMENT OF HYSTERIA.

DR. LUCAS contributes a case of hysteria which may give us pause, to the Glasgow Medical Journal for August, 1830. His object in communicating the case to our contemporary is rather singular for the candid manner in which it is avowed, for he considers it, he says, the bounden duty of a Scotchman to support, as far as he can, every periodical medical publication emanating from the Scotch press. We had thought that the more liberal and distinguished natives of the land of cakes, had begun to disclaim that selfish and too-national feeling which Dr. Lucas openly expresses.

The subject of the case was a stout single woman, 40 years of age, who was seized, in December last, with a violent hysterical attack. It assumed the form of the most excruciating pains referred to the scrobiculus cordis, the patient perspiring profusely, and uttering dreadful cries. Her pulse was full, strong, and rather hard; the attack had come on quite suddenly. The Doctor bled her to 40 ozs. from as large an orifice as he could make, but without relief. He then gave her, in the course of 20 minutes or less, six drachms of laudanum in warm peppermint water, when she began to eructate, and suddenly exclaimed 'I am quite well.' The laudanum only produced an intoxicating effect, but was followed by some nausea for two or three days. Dr. Lucas purged her, and gave her an emetic which operated beneficially, but on the third day she was seized with another hysterical paroxysm like the first. Dr. Lucas's friend, imitating his Pythias, gave the woman three large tea-spoons full of laudanum, and three pills of solid opium, each weighing two grains, in the space of ten minutes. The paroxysm was removed, but others continued to recur till a large blister dispersed them for some time. In another paroxysm Dr. Lucas gave her four drachms of laudanum in the space of 15 minutes, but the cure was produced by tonics and port wine. Now really we think that the treatment was a vast deal too violent, and had the patient been a London female, instead of a stout Glasgow lassie, we doubt whether Dr. L. would have witnessed more paroxysms than one. The bleeding was altogether unnecessary, and the doses of laudanum were much too large. We cannot but think it ridiculous to crowd drachm after drachm of powerful medicines into the human stomach, before the first doses can have had sufficient time to produce their effect. Thus, in 15 minutes, Dr. Lucas's friend gave three large tea-spoons full of laudanum and three pills of solid opium. Had he possessed a little patience it is probable that a drachm or two of laudanum given in an ether or camphor draught, would have answered just as well without any of the risk attendant on the ultra treatment. We do not speak on speculation, for we have witnessed quite as violent cases as this of Dr. Lucas's, but never did we see, nor do we ever expect to see, such violent measures required.

